# Pulmonary Mycobacterium avium-intracellulare is the main driver of the rise in non-tuberculous mycobacteria incidence in England, Wales and Northern Ireland, 2007–2012

**DOI:** 10.1186/s12879-016-1521-3

**Published:** 2016-05-06

**Authors:** Neeraj M. Shah, Jennifer A. Davidson, Laura F. Anderson, Maeve K. Lalor, Jusang Kim, H. Lucy Thomas, Marc Lipman, Ibrahim Abubakar

**Affiliations:** Division of Asthma, Allergy and Lung Biology, King’s College London, London, UK; TB Section, Centre for Infectious Disease Surveillance and Control, Public Health England, London, UK; MRC Clinical Trials Unit and Centre for Infectious Disease Epidemiology, University College London, London, UK; UCL Respiratory, Division of Medicine, University College London, London, UK; 49 Bodley Road, New Malden, Surrey, KT3 5QD UK

**Keywords:** Nontuberculous mycobacteria, Mycobacterium avium-intracellulare complex, Incidence, Environmental mycobacterium

## Abstract

**Background:**

The incidence of non-tuberculous mycobacteria (NTM) isolation from humans is increasing worldwide. In England, Wales and Northern Ireland (EW&NI) the reported rate of NTM more than doubled between 1996 and 2006. Although NTM infection has traditionally been associated with immunosuppressed individuals or those with severe underlying lung damage, pulmonary NTM infection and disease may occur in people with no overt immune deficiency.

Here we report the incidence of NTM isolation in EW&NI between 2007 and 2012 from both pulmonary and extra-pulmonary samples obtained at a population level.

**Methods:**

All individuals with culture positive NTM isolates between 2007 and 2012 reported to Public Health England by the five mycobacterial reference laboratories serving EW&NI were included.

**Results:**

Between 2007 and 2012, 21,118 individuals had NTM culture positive isolates. Over the study period the incidence rose from 5.6/100,000 in 2007 to 7.6/100,000 in 2012 (*p* < 0.001). Of those with a known specimen type, 90 % were pulmonary, in whom incidence increased from 4.0/100,000 to 6.1/100,000 (*p* < 0.001). In extra-pulmonary specimens this fell from 0.6/100,000 to 0.4/100,000 (*p* < 0.001).

The most frequently cultured organisms from individuals with pulmonary isolates were within the *M. avium-intracellulare* complex family (MAC). The incidence of pulmonary MAC increased from 1.3/100,000 to 2.2/100,000 (*p* < 0.001). The majority of these individuals were over 60 years old.

**Conclusion:**

Using a population-based approach, we find that the incidence of NTM has continued to rise since the last national analysis. Overall, this represents an almost ten-fold increase since 1995. Pulmonary MAC in older individuals is responsible for the majority of this change.

We are limited to reporting NTM isolates and not clinical disease caused by these organisms. To determine whether the burden of NTM disease is genuinely increasing, a standardised approach to the collection of linked national microbiological and clinical data is required.

## Background

The frequency of isolation of nontuberculous mycobacteria (NTM) from human clinical samples is increasing worldwide [1]. In England, Wales and Northern Ireland (EW&NI), the incidence of NTM in all sample types was previously reported to have almost trebled between 1995 (0.9 per 100,000 population) and 2006 (2.9 per 100,000 population) [2].

Unlike *Mycobacterium tuberculosis*, which is a genuine pathogen, the largely environmental NTM have been often associated with conditions of host impaired immunity, such as primary immunodeficiency, HIV or the use of immunosuppressive medication [3]. Increasingly, NTM are isolated in immunocompetent individuals, with or without pre-existing structural lung damage [4]. It is not clear why this is so, and possible explanations include an increase in the number of individuals with structural lung damage making them more susceptible to mycobacterial infection, more mycobacteria in the environment, improved laboratory detection techniques, a greater awareness of their potential relevance - resulting in more frequent mycobacterial culture being performed, or the result of more investigation to exclude *M. tuberculosis*.

Here, using population-based data, we report the incidence of NTM isolation in EW&NI between 2007 and 2012. We demonstrate that whilst there has been a significant increase in a number of NTM, the overall rise is largely explained by pulmonary *Mycobacterium avium-intracellulare* complex.

## Methods

All culture positive NTM isolates between 2007 and 2012 reported to Public Health England (PHE) by the five mycobacterial reference laboratories serving EW&NI were included. Age and sex were routinely collected, along with the site of the specimen and NTM species identified from the culture. Between 2007 and 2012, *M. avium, M. intracellulare* and other related organisms, (eg *M. chimaera)*, were grouped as *M. avium-intracellulare* complex (MAC). The rapid growers within *M. abscessus* family (eg *M. abscessus ssp. abscessus*, *M. abscessus ssp. bolletii*, and *M. abscessus ssp. massiliense)* were identifed as *M. abscessus*.

Isolates were probabilistically de-duplicated using patient identifiers to report each individual once [5], based on their earliest specimen date between 2007 and 2012. If an individual had multiple NTM organisms isolated, the organism with the earliest specimen date was included. The site of infection was inferred from the specimen site.

The annual incidence of NTM was calculated using the de-duplicated individual level NTM data and mid-year population estimates from the Office of National Statistics. A chi-squared test for trend was used to analyse the change in incidence between 2007 and 2012. Confidence intervals for incidence were calculated assuming a Poisson distribution for the number of events. All analyses were conducted using Stata 13.1, Stata Corporation, Texas, USA. Incidence rates are expressed per 100,000 population. Means are reported with standard deviation.

## Results

**Fig. 1:**
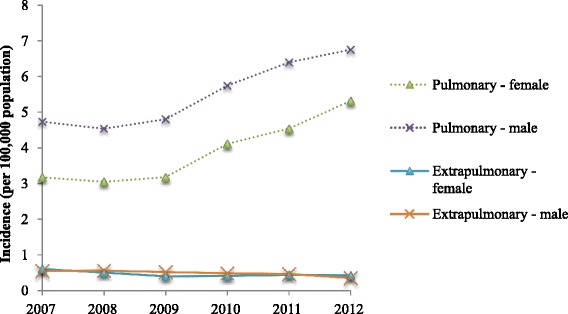
Incidence of isolation of NTM in England, Wales and Northern Ireland 2007–2012

Between 2007 and 2012, 21,118 individuals had NTM culture positive isolates in EW&NI. The overall incidence of NTM increased from 5.6/100,000 (*n* = 3,126, 95 % CI 5.4–5.7) to 7.6/100,000 (*n* = 4,454, 95 % CI 7.4–7.9) (*p* < 0.001; Fig. 1). 16.2 % of individuals had cultures positive for *M. gordonae*. As this is considered non-pathogenic in much of the literature, the rates were recalculated without these isolates. Again the incidence rose (from 4.8/100,000 (*n* = 2,718, 95 % CI 4.6–4.9) to 6.3/100,000 (*n* = 3,651, 95 % CI 6.2–6.5), *p* < 0.001). During the study period 46 different mycobacterial species were identified. More than one NTM species was isolated in 2,100 (10 %) individuals. Species could not be identified in 2.4 %.

Of those with a known specimen site (85.0 %, *n* = 17,932), 90.9 % (*n* = 16,294) had a pulmonary isolate. Fifty-eight percent of these were in men; and the mean (± standard deviation) age of the pulmonary population was 60 ± 20 years. This compares to 53 ± 25 years in those with extra-pulmonary isolates. Ages are expressed as age ± standard deviation.

In people with pulmonary isolates, the incidence rose from 4.0/100,000 (*n* = 2,241, 95 % CI 3.8–4.1) to 6.1/100,000 (*n* = 3,551, 95 % CI 5.9–6.3, *p* < 0.001). In contrast, over the same period it fell in individuals with extra-pulmonary isolates (0.6/100,000, 95 % CI 0.58–0.63 to 0.4/100,000, 95 % CI 0.39–0.41, *p* < 0.001). 16.6 % of individuals with pulmonary isolates had cultures positive for *M. gordonae*. As above, incidence rates were recalculated without these isolates. Incidence rose from 3.4/100,000 (*n* = 1,893, 95 % CI 3.3–3.5) to 5.0/100,000 (*n* = 2,905, 95 % CI 4.9–5.1, *p* < 0.001).

## Organisms in pulmonary samples

**Fig. 2:**
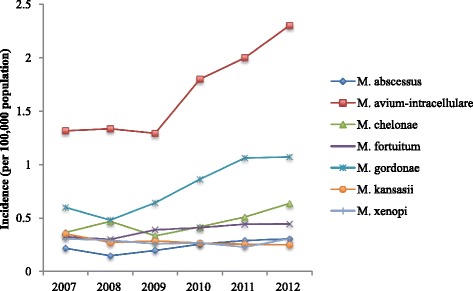
Most frequently isolated NTM organisms in pulmonary samples, 2007–2012

The most frequent NTM cultured from pulmonary samples was *M. avium-intracellulare* complex (MAC; 35.6 %, *n* = 5,800)*.* Other organisms commonly isolated were *M. gordonae* (16.7 %), *M. chelonae* (9.6 %), *M. fortuitum* (8.2 %), *M. kansasii* (5.9 %), *M. xenopi* (5.9 %), and *M. abscessus* (5.0 %) (Table 1 and Fig. 2).

**Table 1:** The relationship between NTM and their isolation from different specimen sites

Organism	Total	Pulmonary		Blood		Lymph node		Urine		Other		Unknown	
	n	n	%	n	%	n	%	n	%	n	%	n	%
M. avium-intracellulare	7436	5800	35.6	120	30.7	161	62.6	65	18.4	187	29.4	1103	34.6
M. gordonae	3414	2721	16.7	2	0.5	2	0.8	99	28.0	33	5.2	557	17.5
M. chelonae	2342	1574	9.7	130	33.2	28	10.9	53	15.0	153	24.1	404	12.7
M. fortuitum	1698	1330	8.2	33	8.4	5	1.9	38	10.7	52	8.2	240	7.5
M. kansasii	1131	966	6.0	2	0.5	4	1.6	12	3.4	32	5.0	115	3.6
M. xenopi	1069	958	5.9	1	0.3	2	0.8	8	2.3	10	1.6	90	2.8
M. abscessus	992	812	5.0	14	3.6	7	2.7	1	0.3	24	3.8	134	4.2
M. malmoense	931	718	4.4	2	0.5	38	14.8	5	1.4	29	4.6	139	4.4
M. peregrinum	489	414	2.5	11	2.8	2	0.8	16	4.5	9	1.4	37	1.2
M. mucogenicum	258	161	1.0	58	14.8	1	0.4	7	2.0	11	1.7	20	0.6
M. marinum	166	1	0.01	0	0	0	0	0	0	75	11.8	90	2.8
Other	1192	839	5.1	18	4.6	7	2.7	50	14.1	21	3.3	257	8.1

“Other” in specimen site category includes specimens from bone, cerebrospinal fluid, eyes, faeces, gastrointestinal tract, peritoneal dialysis fluid, peritoneal fluid, skin and synovial fluid

“%” refers to percentage of NTM species contributing to total for given body site

**Fig. 3:**
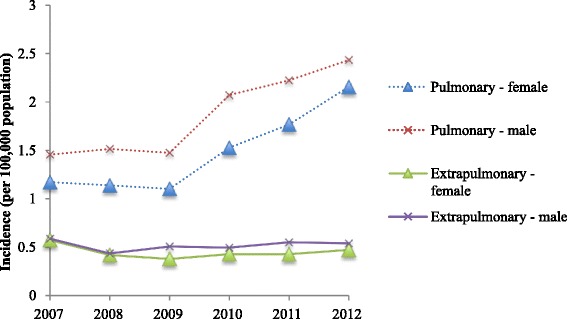
The incidence of *M. avium-intracellulare* isolation, 2007–2012

**Fig. 4:**
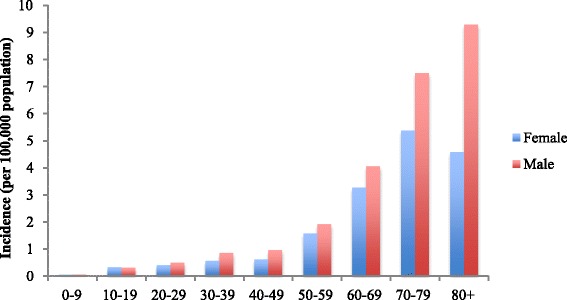
The incidence of *M. avium-intracellulare* in pulmonary samples, by age-group, across all years of this study

The incidence of individuals with pulmonary MAC isolates increased from 1.3 (95 % CI 1.2–1.4) in 2007 to 2.2 (95 % CI 2.1–2.4) in 2012 (*p* < 0.001). This increase was observed in both males and females (Fig. 3). The majority of pulmonary MAC was isolated from older individuals – with around two-thirds of men and women being aged over 60 (Fig. 4). There was also a significant increase in incidence of *M. chelonae, M. fortuitum* and *M. gordonae* (*p* < 0.001); whilst that of *M. kansasii* fell (*p* < 0.001).

**Fig. 5:**
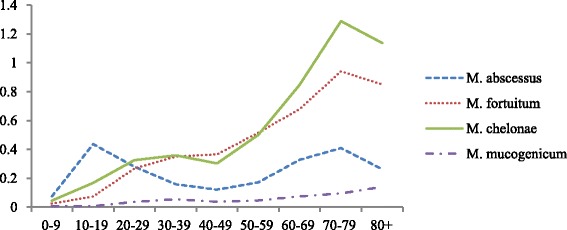
Incidence of rapidly-growing NTM in pulmonary samples, grouped by age across all years 2007–2012

*M. abscessus* had a bi-phasic age distribution with peaks within the age groups 10–19 and 70–79 years. This was unlike other rapid growers such as *M. fortuitum* and *M. chelonae*, where the incidence rose steadily with age (Fig. 5).

## Organisms in extra-pulmonary samples

The most frequent organisms cultured from extra-pulmonary samples were *MAC* (34 %), *M. chelonae* (15.8 %) and *M. gordonae* (14.3 %) (Table 1). Between 2007 and 2012, there was no significant change in incidence in extra-pulmonary MAC isolates (Fig. 3). Unlike other specimen sites, individuals with cultures from lymph nodes were younger, with a mean age of 21 ± 25 years. In these individuals, the most frequent organisms were MAC (62.7 %), *M. malmoense* (14.8 %) and *M. chelonae* (10.9 %). *M. marinum* accounted for 28.7 % of cutaneous isolates.

## Discussion

This population-based study demonstrates that the NTM incidence in submitted samples from EW&NI has continued to rise since the last national investigation [2]. Much of this appears to be due to an increase in NTM in pulmonary isolates, most frequently obtained from older individuals. MAC was the commonest group of cultured organisms from both pulmonary and extra-pulmonary sites. It is noteworthy that the incidence of extra-pulmonary isolates decreased over the same time period over the same time period.

The observed rise in the incidence of NTM in EW&NI is in line with reports from other countries [6, 7], though not Scotland, where there has been no change between 2000 and 2010 [8]. Our finding of pulmonary MAC being the largest contributor to NTM incidence is consistent with a population-based study from Oregon, USA [6].

*M. abscessus* demonstrated a spike in incidence in both younger and older patients, with the majority of isolates found in individuals less than 30 years. Due to the lack of clinical data, we cannot relate this directly to cystic fibrosis (CF), though it is likely that this contributes to the isolates obtained from the younger population where it is an important clinical problem [9]. This is consistent with a recent report from Scotland [8].

*M. chelonae* and *M. mucogenicum* were associated with bloodstream infections. 70 % of cutaneous isolates were *M. marinum* or rapidly growing NTM, which has also been noted by others [10].

There are several possible explanations for the increase in NTM isolation. These include recent environmental changes leading to more NTM in soil or water [3]. In support of this, Khan et al. [11] demonstrated a significant increase in skin sensitization to *M. intracellulare* in the general population in the United States between 1971 and 1972 and 1999–2000. A true rise in the amount of NTM in the environment should also be reflected by a similar increase in extra-pulmonary cultures. However this has actually declined, suggesting that environmental factors are not the only explanation.

More individuals may now be susceptible to pulmonary NTM colonisation and disease. Older males, who were most likely to have a positive NTM culture, are a population with more chronic respiratory disease requiring prescribed drug therapies (including inhaled corticosteroids) that increase the risk of NTM lung disease [12]. Furthermore, the number of people using immunosuppressive and biological agents is rising. Brode et al. [13] reported that anti-TNF therapy doubled the risk of NTM disease (OR 2.19, 95 % CI 1.10–4.37). Also, a patient’s underlying clinical illness may be relevant. For example, rheumatoid arthritis has itself been associated with NTM disease [14, 15].

Clearly these explanations are not mutually exclusive; and over time could lead to a yet further increase in incidence as clinicians become more aware of NTM disease and so perform more mycobacterial diagnostic tests.

A strength of our study is that we are confident we are counting only single isolates from individuals, and hence avoiding duplication (and inflation) of our results. The natural history of NTM (where an individual will isolate often multiple samples over an extended period of time) means that this is a common problem when dealing with this condition [2]. We believe that the absolute number of NTM isolates being reported to Public Health England has genuinely increased. However as only the earliest culture result was included in our study in those individuals who had positive cultures of more than one NTM organism (10 %), there is a risk we may have introduced bias towards rapid growers at the expense of slower growers. Despite this, the predominant NTM was MAC, a slow-growing group of organisms.

To determine whether the burden of NTM disease is also increasing requires an understanding of current clinical and laboratory practice. In EW&NI there is no standardised approach to the investigation of suspected NTM patients, and the systematic collection of relevant clinical data related to NTM isolates is limited. This needs to change if we are to better understand NTM disease and improve on current management strategies and outcomes.

## Conclusion

The continuing rise in the isolation of NTM between 2007 and 2012, driven primarily by pulmonary MAC in older individuals warrants further investigation. It is now imperative that standardised clinical data are collected on all individuals with positive NTM isolates across the country to determine if there is a genuine increase in NTM disease. This will allow determination of clinical risk groups and reasons for this increase, and justify targeting the development of the management of pulmonary MAC.

## Abbreviations

CF: cystic fibrosis
EW&NI: England, Wales and Northern Ireland
NTM: nontuberculous mycobacteria
PHE: Public Health England
